# No evidence for learned mating discrimination in male *Drosophila pseudoobscura*

**DOI:** 10.1186/1471-2148-6-54

**Published:** 2006-07-06

**Authors:** Nikolai P Kandul, Kevin M Wright, Ekaterina V Kandul, Mohamed AF Noor

**Affiliations:** 1 DCMB Group, Department of Biology, Box 91000, Duke University, Durham, NC 27708, USA

## Abstract

**Background:**

Since females often pay a higher cost for heterospecific matings, mate discrimination and species recognition are driven primarily by female choice. In contrast, frequent indiscriminate matings are hypothesized to maximize male fitness. However, recent studies show that previously indiscriminate males (e.g., *Drosophila melanogaster *and *Poecilia reticulata*) can learn to avoid heterospecific courtship. This ability of males to discriminate against heterospecific courtship may be advantageous in populations where two species co-occur if courtship or mating is costly.

**Results:**

Here, we tested whether *Drosophila pseudoobscura *males learn to discriminate against heterospecific females after being exposed to and rejected by *D. persimilis *females. In most of our assays, we failed to observe differences in *D. pseudoobscura *courtship intensity of heterospecific females by males that had previously courted heterospecific females vs. males that had been maintained in isolation.

**Conclusion:**

We conclude that learning to avoid heterospecific courtship may not be universal, even within the genus Drosophila, and may possibly be dependent on the natural history of the species.

## Background

Preferentially mating with one's own species (behavioral isolation) is an important barrier to gene flow separating many animal species [1]. The complex behaviors associated with mating allow organisms to choose the best mate and/or to distinguish between species. The evolution of these behaviors is hypothesized to be driven by female choice because of the larger reproductive investment into eggs relative to sperm. A female with a limited number of eggs will maximize her fitness by producing viable, fertile progeny, and should therefore strongly prefer to mate with a conspecific male. In contrast, males often invest little in reproduction and are thus predicted to be less discriminate with respect to their mating partners; their fitness will be maximized by mating with as many females as possible, and the costs of mating with heterospecifics may be much less. However, these oversimplifications ignore male investment of time and effort into courtship rituals that either result in no mating or in sterile progeny. If the courtship or reproductive investment is substantial [e.g. nuptial gifts in insects [2]], or if time for mating is limited because of a short reproductive window, then selection would also increase male mating discrimination.

Still, the reigning paradigm in studies of reproductive behavior is that males are often indiscriminate in mating [3]. This paradigm has been most recently challenged by innovative experiments testing for the ability of males to *learn *to avoid heterospecific courtship after repeated exposure to heterospecific females [4,5]. Trinidadian guppies (*Poecilia reticulata*) living in allopatry show little male mating discrimination, but upon exposure to their sister species, *P. picta*, quickly learn to avoid interspecific crosses [5]. This learned discrimination appears to have become assimilated in sympatric populations [6]. Similarly, *Drosophila melanogaster *males were originally described as indiscriminate [e.g. [7]]. However, Dukas [4] found that *D. melanogaster *males exposed to *D. simulans *females display significantly fewer mating attempts with heterospecifics than males not exposed to heterospecific females. Thus putatively indiscriminate males can learn to avoid heterospecific courtship, and they imply that there is a cost to indiscriminate male courtship or mating. These findings suggest that male species discrimination may be very common but have been missed because of unnatural laboratory mating designs (i.e., exclusive use of naïve males never exposed to females before).

We test the generality of learned male mating discrimination by examining a pair of North American fruit flies, *D. pseudoobscura *and *D. persimilis*. Males of these species have been shown previously to court *D. pseudoobscura *and *D. persimilis *females at equal frequencies in several types of both choice and no-choice experiments [8,9], but all of these experiments employed naïve males. Matings between these two species result in fertile females and sterile males; thus there may be some cost of even successful interspecies mating for males. Nonetheless, F_1 _hybrids have been collected from nature [10], and a molecular signature of interspecies introgression has been documented [e.g. [11]].

If the findings of Dukas [4] in *D. melanogaster *are general to the genus, we predict that *D. pseudoobscura *males will be more reluctant to court heterospecific females after being exposed to and rejected by a heterospecific female. Such discrimination may be more apparent in strains derived from sympatric versus allopatric populations, as has been described for females of these species [12].

## Results

If *D. pseudoobscura *males learn to avoid courting heterospecifics after earlier exposure, we predict that the "experienced" males should exhibit 1) a longer courtship latency, 2) fewer courtship wing vibrations, and 3) fewer attempted copulations than the "naïve" males. We also note that, in all of our experiments, male courtship was both intense and consistent, hence providing no obvious indication that males were at all reluctant to court heterospecific females.

### First mating assays

No significant differences were observed between naïve and experienced males in courtship latency, frequencies of wing vibrations, or attempted copulations in assays using the Flagstaff, Arizona, strain (Table 1). For assays with the Mather, California strain, the experienced males exhibited reduced courtship intensity, as measured by frequencies of wing vibrations and attempted copulations (*P *= 0.012 and *P *= 0.027, respectively), and mating success relative to the naïve males (Fisher's exact test, *P *< 0.01). However, this finding may be biased from the exclusion of a large fraction (40%) of males from the experienced group that mated in their prior experience. Hence, those males that are most vigorous and most attractive to heterospecific females have been taken from the pool of experienced males, but not from the pool of naïve males, prior to the experimental treatment.

**Table 1:** Courtship intensity of *D. pseudoobscura *males toward *D. persimilis *females

Treatment	N	Courtship latency	Frequency of time spent in Wing Vibrations	Frequency of time Attempting Copulations	Matings
(1) 8-day old flies used for exposure treatment and courtship assays.					
Flagstaff, Arizona (50% males were excluded after experience treatment)					
Experienced	34	52.7 (4)	0.039	0.038	10
Naïve	34	90.1 (5)	0.040	0.028	13
Mather, California (40% males were excluded after experience treatment)					
Experienced	82	58.6 (4)	0.025*	0.017*	14*
Naïve	82	64.9 (3)	0.037	0.024	31

(2) 3-day old flies used for exposure treatment and courtship assays.					
Flagstaff, Arizona (10% males were excluded after experience treatment)					
Experienced	74	36.7 (0)	0.036	0.011	2
Naïve	74	34.9 (0)	0.033	0.013	4
Mather, California (10% males were excluded after experience treatment)					
Experienced	74	55.2 (2)	0.022*	0.007	2
Naïve	74	56.7 (5)	0.017	0.006	2

(3) 8-day old males were confined with 3-day old females, and then the experienced males courted 8-day old females.					
Flagstaff, Arizona (10% males were excluded after experience treatment)					
Experienced	73	35.2 (1)	0.119	0.070	49
Naïve	73	52.5 (0)	0.125	0.068	51
Mather, California (20% males were excluded after experience treatment)					
Experienced	62	41.4 (0)	0.096	0.063	34
Naïve	62	35.6 (1)	0.089	0.054	40

Means of courtship latency (in seconds), frequencies of wing vibrations and of attempted copulations (as numbers per seconds), and number of matings are presented in the table. Males that never courted were not included in the estimates of mean courtship latency, and their numbers are shown parenthetically. Mann-Whitney U and Fisher's exact tests were used to estimate the statistical significance. * P < 0.05.

### Second mating assays

No significant differences were observed between the naïve and experienced males in courtship latency, frequency of attempted copulation, or number of matings (Table 1). In the Mather, California, strain, the experienced males elicited slightly more frequent wing vibrations on average than the naïve males (*P *= 0.047), but this observation is *opposite *in direction to the prediction of experienced males learning to avoid courting heterospecifics.

### Third mating assays

No significant differences were observed between naïve and experienced males in any parameters used to measure the courtship intensity (Table 1).

## Discussion

Across most of our mating experiments, we did not observe differences in *Drosophila pseudoobscura *courtship intensity of heterospecific females by males that had previously courted heterospecific females vs. males that had been maintained in isolation. Hence, we failed to find evidence that *D. pseudoobscura *males learn to avoid futile courtship of heterospecific females after being previously rejected by them. These findings contrast similar studies in *D. melanogaster *[4] and guppies [5] and suggest that the process of male learning to avoid courting heterospecifics is not universal. These findings also further evince that *D. pseudoobscura *males are indiscriminate of species identity in their courtship of females [see also [8,13]].

In one set of experiments, the naïve males of the Mather, California, strain displayed significantly higher intensity of courtship, as measured by frequencies of wing vibration and attempted copulation, and mating success than the experienced males in the first mating assays. However, we interpret this to be an artifact of the removal of a large fraction of males that are most vigorous and most attractive to *D. persimilis *females from the experienced group (see Results), as this difference was not apparent (and in fact, opposite in direction) in assays of the same strain when fewer males were excluded. The same phenomenon may have also operated in 8-day posteclosion males from the Flagstaff, Arizona, strain (experiment 1), but the smaller sample size of those males tested likely rendered the result statistically nonsignificant (see Table 1). The high mating propensity of older flies [e.g. [14,15]] contributed to this artifact. Although our three mating assays cannot be statistically compared because they were not temporally controlled, our results seem to confirm the published findings of lower receptivity of young females (see Table 1). Hence, we interpret our results from the second and third mating assays to be generally less biased for evaluating the hypothesis of learned mating discrimination, since a much smaller fraction of (likely more vigorous) males were removed. The only statistically significant difference observed in these latter mating assays was opposite to that predicted by the hypothesis that males learn to avoid fruitless courtship of heterospecific females (i.e. the experienced males of the Mather, California, strain elicited a higher frequency of wing vibrations than the corresponding naïve males in the second mating assays).

Some differences seem apparent in various parameters between our six experiments (two lines × three treatments), such as possible differences between the Flagstaff and Mather lines in courtship latency in our second experiment. However, we stress that Drosophila courtship is labile to subtle environmental factors, and comparisons between experiments that were not temporally controlled are suspect. For the conclusions we present here, we focus only on temporally controlled studies where exactly equal numbers of flies were tested at the same times on the same days on the same batch of media, etc.

In our experiment, we did not exactly replicate the conditions used by Dukas [4] in his study of *D. melanogaster*, but our changes in design were done to better simulate the natural history differences between *D. pseudoobscura *and *D. melanogaster*. Unlike *D. melanogaster*, courtship in *D. pseudoobscura *is very rapid, and females appear to evaluate males individually based on a sequential encounter model [16]. As a result, we shortened the exposure treatments in our study and also used single males and females. While some of these design differences may affect the comparability of our results to those of Dukas [4], we argue that our approach more accurately reflects (to the best of our knowledge) the behavior of *D. pseudoobscura *in nature.

The evolutionary reason for a potential difference in male learning ability among *Drosophila *species is uncertain. *D. pseudoobscura *males should encounter some cost through futile courtship of heterospecific females, and even if mating would be successful, half the F_1 _progeny would be sterile. This mismating cost has putatively driven the evolution of greater female discrimination in this species [12]. One could speculate, however, that courtship is much shorter or less energy-intensive in *D. pseudoobscura *than in *D. melanogaster*, which would be consistent with the very short mating latencies observed in the laboratory [8] and the field [16] in the former species. Alternatively, a high level of 7-tricosene, a known anti-aphrodisiac for *D. melanogaster *males [17], in the cuticular hydrocarbon profile of *D. simulans *virgin females [18] can serve as a strong conditioning agent of male behavior in *D. melanogaster *[19]. 7-tricosene is the major component of the cuticular hydrocarbon profile of *D. melanogaster *males [20]. It is remarkable that the concentration of 7-tricosene increases in *D. melanogaster *mated females, and hence renders them unattractive to males [17,20]. Both virgin females of *D. pseudoobscura *and *D. persimilis *have a slightly higher proportion of 2-methyl hexacosane (2-MH) in their cuticular hydrocarbon profiles [21] than males, and hence *D. persimilis *may not provide a strong conditioning agent that can render male behavior in *D. pseudoobscura*. Finally, there may simply be less detectable phenotypic difference between *D. pseudoobscura *and *D. persimilis *than between *D. melanogaster *and *D. simulans*, which is consistent with human observations of these species pairs.

Certainly, our assays did not mimic the natural setting perfectly, and available data on reproductive ecology of *D. pseudoobscura *and *D. persimilis *in natural populations are limited. Hence, we cannot completely exclude the possibility that male learning to avoid heterospecific courtship does occur in nature and that we could not detect it because of an unknown environmental variable. However, the confinement periods we used were still longer than courtships observed in nature [16]. We also used adult males at two different ages, 3-day and 8-day posteclosion, and the courtship intensity of *D. pseudoobscura *males toward heterospecific females did not change in the second and third mating assays.

Selection should be strongest in species for which males invest large amounts of time into finding mates, courtship, and/or rearing offspring. Thus, the breeding ecology of a species is likely a very influential factor in determining if male discrimination evolves. Studies of *P. reticulata *show that fish living in sympatry with their sister species discriminate against heterospecifics, while those living in allopatry are indiscriminate but quickly learn to avoid matings with heterospecifics [5]. This male discrimination against heretospecific females can become innate in the places where *P. reticulata *co-occur with *P. picta*, and lead to reproductive character displacement between the two species [6]. While we lack basic natural history information in *D. pseudoobscura*, our study suggests the possibility that differences in breeding ecology between *D. pseudoobscura *and *D. melanogaster *may have led to courtship being less costly in *D. pseudoobscura *such that there is no selective pressure for males to discriminate.

## Conclusion

We failed to find evidence that *Drosophila pseudoobscura *males moderate their subsequent courtship of heterospecific females after encountering them and being rejected by them. Although some differences in experimental design were employed to more precisely reflect the natural history of our focal species, these findings nonetheless contrast published findings from studies of *D. melanogaster *[4]. As a result, we conclude that learning to avoid heterospecific courtship does not appear to be universal, even within the genus Drosophila, and may possibly be dependent on the natural history of the species.

## Methods

### Stocks

We tested for the roles of male choice in strains derived from two *D. pseudoobscura *populations: one sympatric with *D. persimilis *(Mather, CA, 17) and one allopatric to *D. persimilis *[Flagstaff, AZ, 1993: both strains are described in [22]]. An earlier study provided evidence for natural selection strengthening female discrimination in sympatric versus allopatric populations, supporting the theory of reinforcement [12]. *D. persimilis *flies were from a strain isolated from Mount St. Helena, CA, in 1993. These strains have been maintained in a constant laboratory environment at 20°C on standard dextrose/yeast/agar medium.

### Experimental design issues

These two species are morphologically identical, and intraspecific courtship proceeds very rapidly, both in the laboratory and in nature, with copulations occurring within a few seconds of courtship initiation [16]. Females do not exhibit any noticeable consistent stereotyped receptivity behavior aside from allowing copulation. Because of this rapid courtship and lack of stereotyped female receptivity behavior, it is difficult to compare intraspecific and interspecific courtship intensities since intraspecific courtship results in immediate copulation in virtually all (>90%) pairings under the conditions used. However, a previous study [8] nonetheless attempted to identify evidence for discrimination in naïve males, and failed to see any under comparable conditions in courtship latency (no-choice), in direction of first courtship (choice), or in courtship intensity of manipulated females that could not mate (choice). The Flagstaff 1993 line was even one of the lines tested in that earlier study. Hence, based on this extensive previous work, we assume that the naïve males we tested are indiscriminate in their courtship, and directly compared naïve and experienced *D. pseudoobscura *males in their courtship of *D. persimilis *females.

### Experiments

Virgin adult male *D. pseudoobscura *and female *D. persimilis *flies were collected within 8 hours of eclosion and maintained in isolation from the other sex until the beginning of the experiments. The day before each mating experiment, males were placed in vials individually to decrease crowding-mediated courtship inhibition [23].

We used 8-day posteclosion flies following Noor [12] for the first set of mating assays, which we consider essentially a "pilot study." For each experiment, *D. pseudoobscura *males were randomly assigned to the naïve or experienced treatments. The experienced treatment group males were placed in a vial with a virgin female *D. persimilis *for the exposure treatment, which lasted 30 minutes. Males courted and attempted mating with the heterospecific female quickly after being placed in the vial and continued to court through most of the exposure period. Very few males (<<10%) failed to court in the exposure period in any of our studies, and those that did fail to court were excluded from subsequent use. All males that successfully mated in the exposure period were removed from the experiment to avoid artifacts associated with possible differences in virgin vs. nonvirgin male behavior. After exposure to a heterospecific female, males were placed in isolation for an additional 30 minutes until the courtship assays began.

Both females and males of *D. persimilis *and *D. pseudoobscura *are known to be more receptive to mating with increasing age [e.g. [14,15]]. To examine whether learning may have been possible in males that were perhaps less "desperate," 3-day posteclosion flies (males and females) were used for the second set of mating assays. Flies are sexually mature at this age and will mate. In this set of experiments, the protocol was otherwise identical except that the exposure treatment lasted 10 minutes, and males spent 10 minutes in isolation before the courtship assays began. We also switched from 30-minute exposures to 10-minute exposures to better simulate behaviors in nature, where courtships are typically very short [16].

In the first set of mating assays, approximately 40% of 8-day posteclosion males mated in their original confinement with heterospecific females, and thus were excluded from the courtship assays (Table 1). This exclusion of a very large fraction of males from the experienced treatment could have biased the comparison between the experienced and naïve group males (see results). To decrease the possible bias created by the exclusion of the most vigorous and/or most attractive males, we paired 8-day posteclosion males *D. pseudoobscura *with 3-day posteclosion *D. persimilis *females in a ten-minute exposure treatment, and then paired the males with 8-day posteclosion females in the courtship assays. By pairing males with less receptive females in the exposure treatment, a smaller fraction were excluded because of mating, reducing the possible bias observed in the first set of experiments. In this third set of mating assays, the exposure treatment again lasted 10 minutes, and males spent 10 minutes in isolation until the courtship assays began.

All courtship assays were performed in a pairwise manner with equal numbers of experienced and naïve males assayed each morning. Single *D. pseudoobscura *males were placed in vials with a single *D. persimilis *female and observed for 10 minutes. Four measures of courtship intensity were recorded: courtship latency, number of wing vibrations, and number of attempted copulations. The number of successful matings was also recorded for each treatment within each population. To account for the males that successfully mated and thus stopped pursuit of females prior to the end of the courtship assays, the frequencies of wing vibrations and attempted copulations (i.e., numbers of wing vibrations and attempted copulations divided by a courtship time in seconds) were used for comparing the experienced and naïve male groups. Sympatric and allopatric populations were scored simultaneously for experiment 2 and during different weeks for experiments 1 and 3. The first experiment was executed approximately one year before the second and third experiments. For particular trials, all mating experiments were performed between 9:00 and 11:00 am each morning.

### Statistics

We tested for statistical differences between experienced and naïve flies on all courtship measures using the nonparametric Mann-Whitney U-test, given the non-normality of the data. We present means rather than ranks in the Table for ease of visualization but not standard errors given the non-normality. The results from the sympatric and allopatric populations and from the three mating assays were analyzed separately. Fisher's exact tests were employed to test for significance in differences in mating success between experienced and naïve flies.

## Authors' contributions

MAFN conceived the study and coordinated its execution. The first mating assays were performed by KMW. The second and third mating assays were performed NPK and EVK. NPK performed the statistical analyses. All authors contributed to preparation of the manuscript.
